# Supplementary material for: Combination of Kaempferol and Docetaxel Induces Autophagy in Prostate Cancer Cells In Vitro and In Vivo
Source: Int J Mol Sci. 2023 Sep 25;24(19):14519. doi: 10.3390/ijms241914519 (PMC10572510; doi:10.3390/ijms241914519)
Supplement: Supplementary file 1 [file ijms-24-14519-s001.zip › ijms-2570063-supplementary.pdf]

Supplementary Material

1.Supplementary Data

1.1 Fig.2B in our manuscript represents the analysis outcomes of the whole uncropped images in Sup.M.Fig.S1.

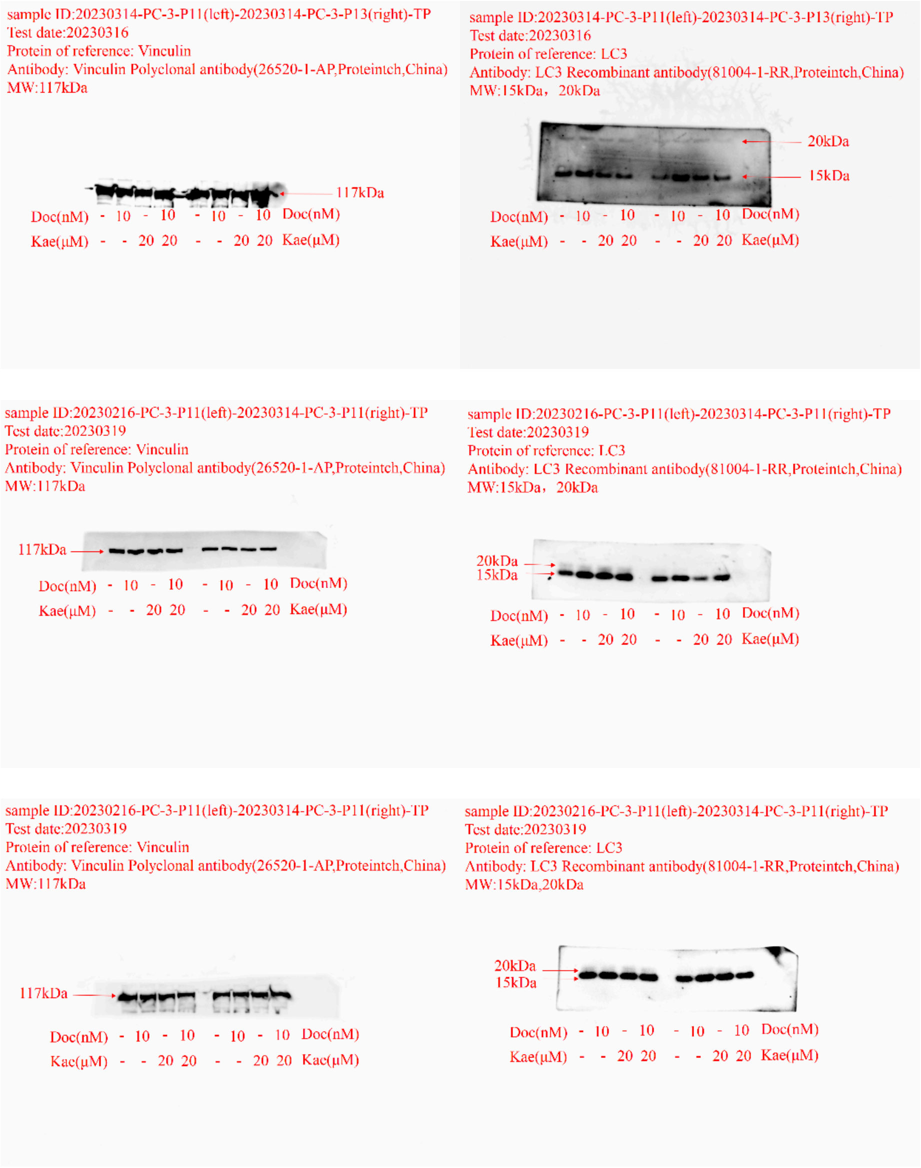

Sup.M.Fig.S1 Original western blots images of LC3.

1.2 Fig.2C in our manuscript represents the analysis outcomes of the whole uncropped images in Sup.M.Fig.S2.

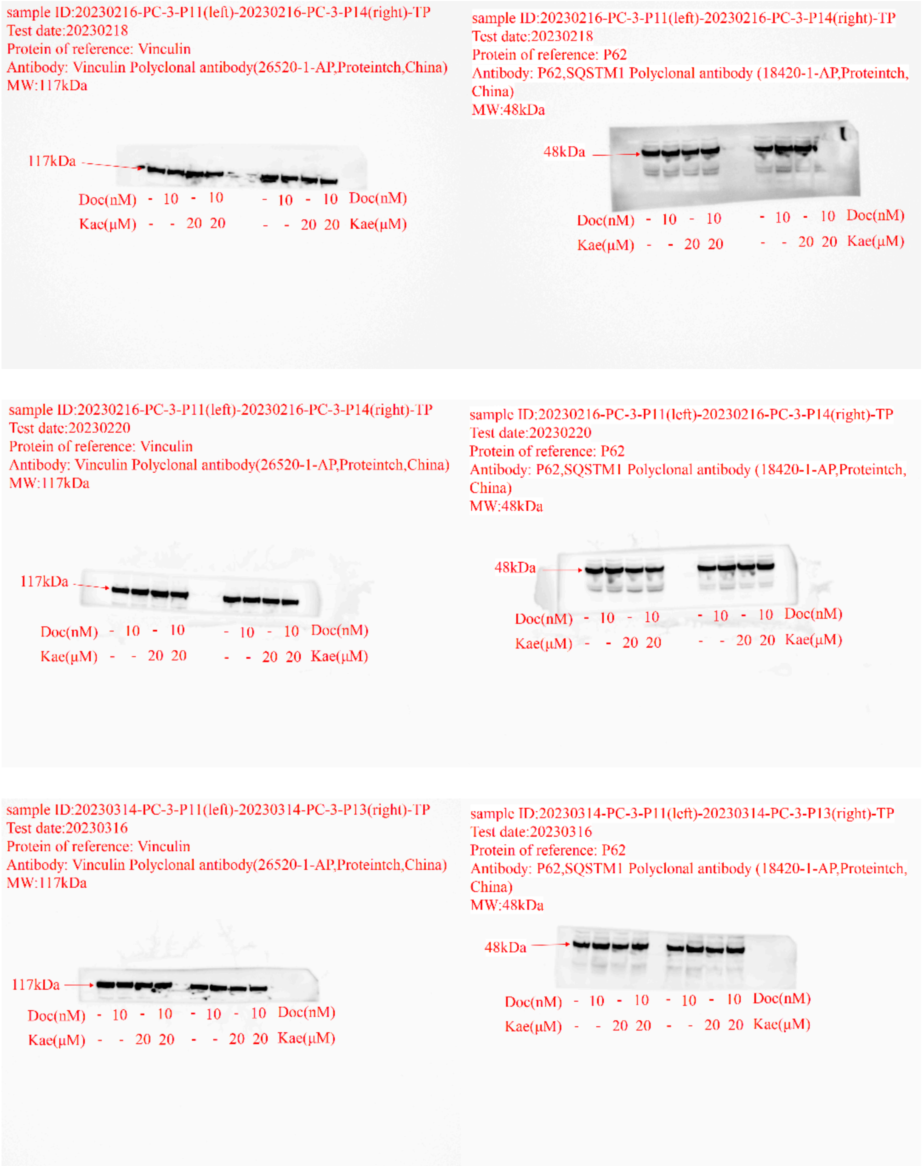

Sup.M.Fig.S2 Original western blots images of P62.

1.3 Fig.6A in our manuscript represents the analysis outcomes of the whole uncropped images in Sup.M.Fig.S3.

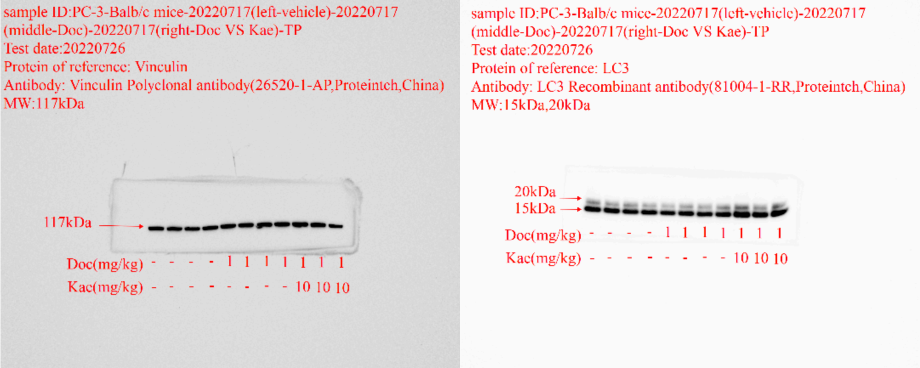

Sup.M.Fig.S3 Original western blots images of LC3.

1.4 Fig.6B in our manuscript represents the analysis outcomes of the whole uncropped images in Sup.M.Fig.S4

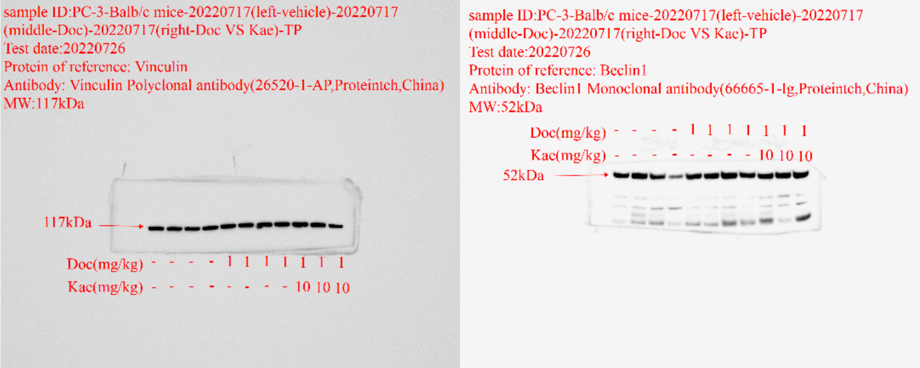

Sup.M.Fig.S4 Original western blots images of Beclin1.

1.5 Fig.6C in our manuscript represents the analysis outcomes of the whole uncropped images in Sup.M.Fig.S5

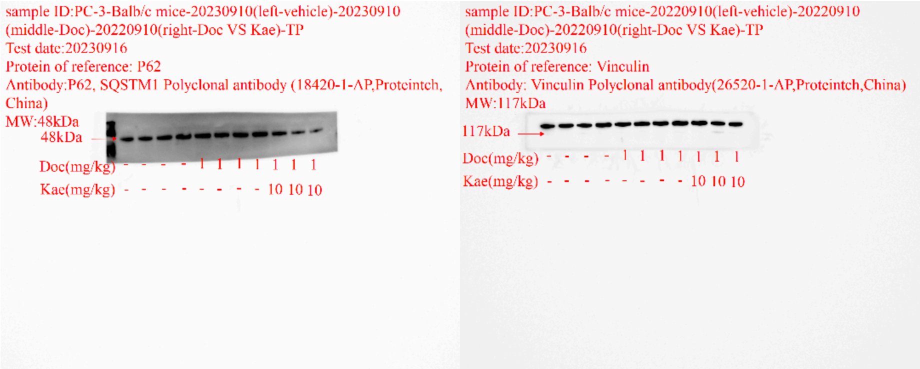

Sup.M.Fig.S5 Original western blots images of p62.

1.6 Fig.6D in our manuscript represents the analysis outcomes of the whole uncropped images in Sup.M.Fig.S6

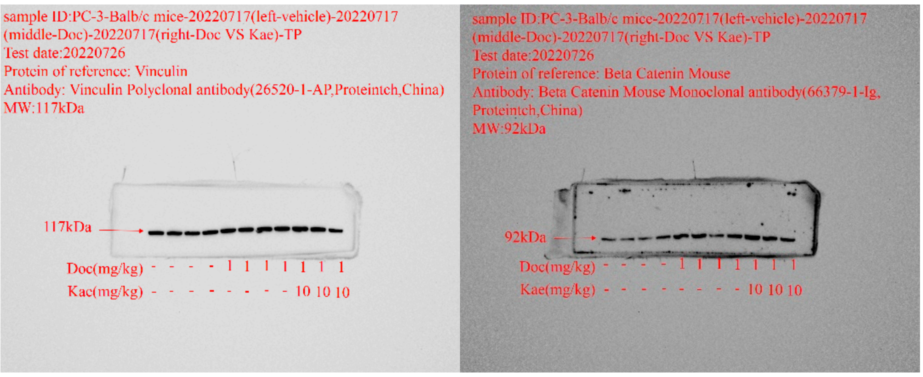

Sup.M.Fig.S6 Original western blots images of Beta catenin.

1.7 Fig.6E in our manuscript represents the analysis outcomes of the whole uncropped images in Sup.M.Fig.S7

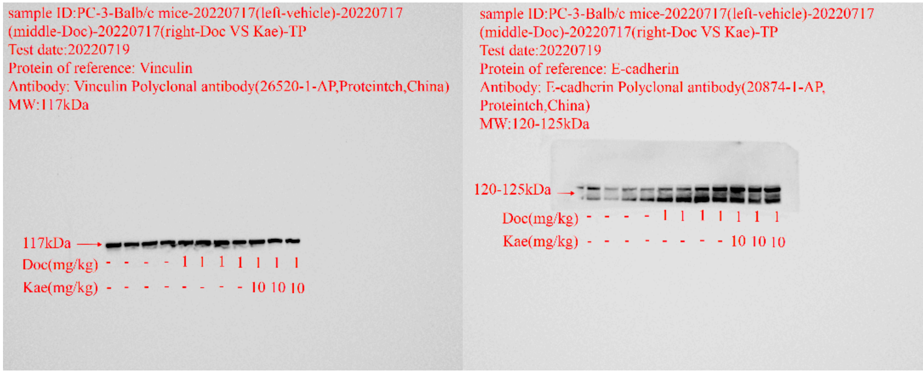

Sup.M.Fig.S7 Original western blots images of E-cadherin.

1.8 Fig.6F in our manuscript represents the analysis outcomes of the whole uncropped images in Sup.M.Fig.S8

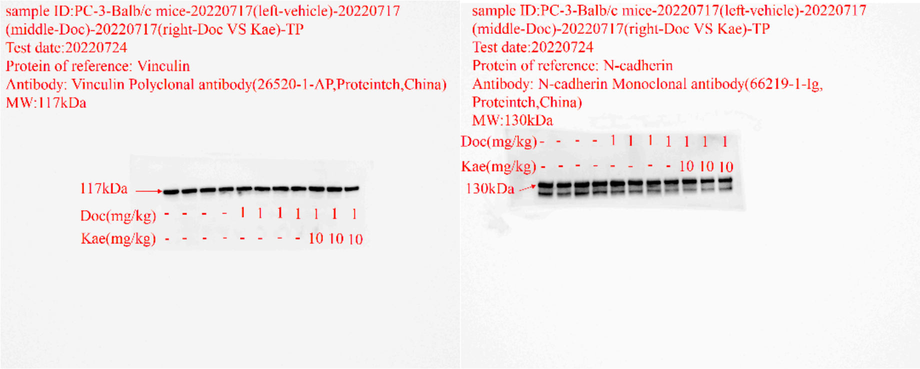

Sup.M.Fig.S8 Original western blots images of N-cadherin.

1.9 Sup.fig.S1A in our manuscript represents the analysis outcomes of the whole uncropped images in Sup.M.Fig.S9

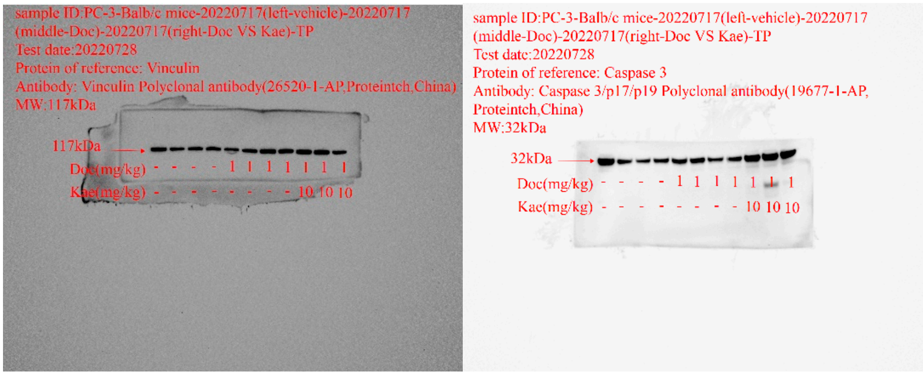

Sup.M.Fig.S9 Original western blots images of Full length-caspase3.

2. Supplementary Figure

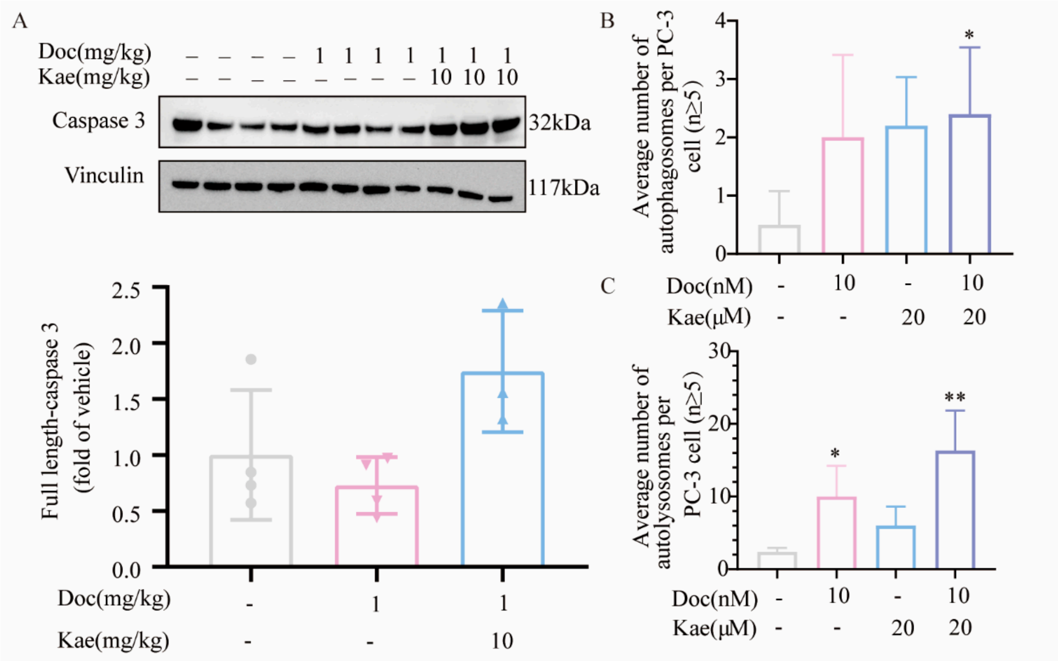

Sup.fig.S1. (A) Full length caspase 3 expression in tumor tissue of nude mice; (B) The quantification of autophagosomes in PC-3 cells; (C) The quantification of autolysosomes in PC-3 cells.
